# Dermal reaction and bigeminal premature ventricular contractions due to neostigmine: a case report

**DOI:** 10.1186/1752-1947-5-83

**Published:** 2011-02-25

**Authors:** Fardin Yousefshahi, Mohammad Anbarafshan, Patricia Khashayar

**Affiliations:** 1 Anesthesia and Critical Care Department, Women Hospital & Tehran Heart Center, Tehran University of Medical Sciences, Tehran, Iran; 2 Anesthesia and Critical Care Department, Sina Hospital, Tehran University of Medical Sciences, Tehran, Iran; 3 General Practitioner, Endocrinology and Metabolism Research Center, Tehran University of Medical Sciences, Tehran, Iran

## Abstract

**Introduction:**

Neostigmine is a frequently used acetylcholinesterase inhibitor administered to reverse muscular relaxation caused by nondepolarizing neuromuscular relaxants in patients recovering from general anesthesia. Severe allergic reactions and urticaria are rarely reported following the use of neostigmine bromide, and never with methylsulfate-containing drugs. In this case, bigeminal premature ventricular contractions added to urticaria provides a warning about the possibility of a life-threatening situation.

**Case presentation:**

We report the case of a 23-year-old Persian woman who presented with bigeminal premature ventricular contractions along with urticarial lesions on her arm and trunk as soon as she was administered neostigmine methylsulfate after undergoing a laparoscopy for ectopic pregnancy.

**Conclusion:**

This case report could be of value not only for anesthesiologists who routinely use neostigmine but also for others who administer the pharmaceutical preparation in other situations. The report presents a rare case of drug reaction following neostigmine use. As a result, one should consider any drug a probable cause of drug reaction. The preparation of resuscitative facilities, therefore, is necessary prior to the prescription of the medication.

## Introduction

Neostigmine, generally used in combination with bromide or methylsulfate, is an acetylcholine esterase inhibitor prescribed mainly to reverse the effects of muscular relaxants at the end of operations performed with the patient under general anesthesia. The drug is also used in patients with myasthenia gravis and paralytic ileus [1-3].

Increased saliva, nausea and vomiting, abdominal cramps, cardiac dysrhythmia and diarrhea are the commonly reported side effects of the drug [4]. Severe allergic reactions and urticaria, however, are rarely reported following the use of neostigmine bromide, and never with methylsulfate-containing drugs [5]. This article presents the case of a pregnant woman who developed a 5 mm wheal on her left forearm after receiving neostigmine during an operation.

## Case presentation

The patient was a 23-year-old Persian woman who weighed 60 kg. She underwent a laparoscopy at the eighth week of gestation (G1Ab0L0) because of a right adenexal mass and free liquid in her dorsal cul de sac space.

Considering the patient's medical record, there was no evidence of any underlying diseases or positive history of allergic reactions to food or drugs in her or her close family. She had never undergone any operations before and had no previous exposure to neostigmine. Additionally, there was no positive finding in her medical history, preoperative examinations and laboratory findings (complete blood count, erythrocyte sedimentation rate, blood urea nitrogen, creatinine and liver function tests).

Midazolam (2 mg) and fentanyl (50 μg) plus thiopental (5 mg/kg), atracurium (0.5 mg/kg) and lidocaine (1 mg/kg) were injected to induce anesthesia. The patient received halothane (1.2 minimum alveolar concentration) and oxygen (100%) for anesthesia maintenance. Two minutes before the surgical incision was performed, 25 μg fentanyl was injected; atracurium (0.15 mg/kg) was then administered every 20 minutes during the operation.

The surgeon suctioned about 500 ml of blood from the patient's abdominal cavity; no blood, however, was transfused during the two-hour, 40-minute operation. The patient's vital signs were monitored throughout the surgery, and no specific complication was reported.

**Figure 1:**
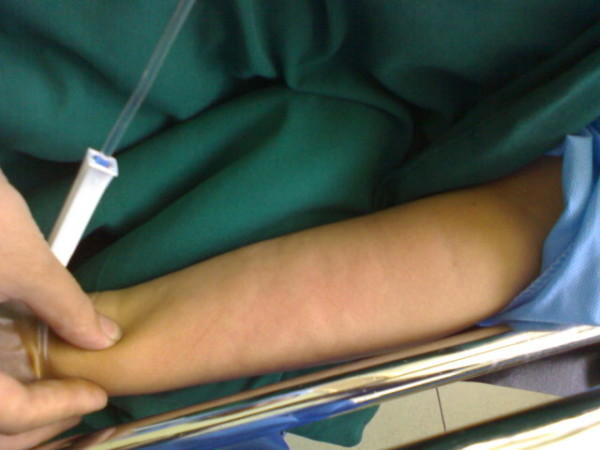
**Urticaria lesions in the recovery room 30 minutes after occurrence, when partial resolution is obvious**.

At the end of the operation, the patient was still in a deep anesthetic stage and did not respond to painful stimuli. Partial muscular force, however, was restored as soon as the inhaled anesthesia was ceased; thereafter neostigmine (0.04 mg/kg) and atropine (0.02 mg/kg) were infused slowly. At this time, the patient developed a 5 mm urticarial lesion along the vein course that quickly spread over the left forearm. The erythematous convex lesions spread in a geographical pattern but were less severe in the neck and chest (Figure 1).

Bigeminate premature ventricular contractions (PVCs) were also noted at the same time but disappeared spontaneously after a few minutes. It should be noted that the patient's heartbeat (80beats/min), blood pressure (110/60 mmHg) and blood oxygen saturation (SpO_2_) (98%) were all normal at this time, and there was no sign of wheeze or other abnormal lung sounds in auscultation.

Hydrocortisone (200 mg) was prescribed for the lesions. The tracheal tube was removed as soon as the patient became conscious, and she was then transferred to the recovery room. No other complications such as the development of dermal lesions or cardiac dysrhythmia were reported during the patient's hospitalization, and she was discharged in good condition.

A sample of the prescribed neostigmine was sent to the laboratory of the Pharmacology Faculty; further analysis revealed the medication to be neostigmine methylsulfate with 102.85% effective substance (in accordance with USP30 reference) with no other additives.

The postsurgical echocardiography was reported to be normal. A skin test performed a few months after the operation revealed a 5 mm wheal and 7 mm flare after the patient was exposed to neostigmine; such a reaction, however, was not noted following the exposure to atropine, normal saline and latex. Additionally, histamine exposure was associated with the development of an 8 mm wheal and a 9 mm flare, suggestive of a positive dermal reaction. It should be noted that the patient did not agree to undergo additive complement component C3 or C4 and antinuclear antibody tests.

## Discussion

Drug reactions presenting as dermal lesions is not a known phenomenon following the use of neostigmine, particularly neostigmine methylsulfate. While the underlying cause of urticaria following allergen exposure may remain unclear in certain cases, the presence of active components, preservatives or conveying combinations (parabens and aldeheids) are often considered the main cause of drug-related urticaria.

In our case, the absence of any preservatives or conveying combinations in the specific compound, along with the results of the performed skin test, supported the hypothesis that the neostigmine molecule itself had been the main cause of the reported allergic reaction.

Arrhythmia, especially PVCs, is common during anesthesia, particularly during the intubation and extubation time, when the blood anesthetic level is lightened and the airway is stimulated. There are many other factors to take into account, including cardiac disease, direct stimulation, toxins and allergens, contributing to arrhythmia during surgery. PVCs are often benign and resolve spontaneously, but rarely are a precursor of life-threatening arrhythmias.

In the present case, the patient was in deep stages of anesthesia when the muscular block was reversed using a combination of neostigmine and atropine. Considering the fact that there was no noxious stimulation at the very moment or minutes before that, the occurrence of the lesion could be considered an obvious drug eruption.

## Conclusion

This case could be of value not only for anesthesiologists, as physicians who administer neostigmine routinely, but also for others who are involved in pharmaceutical preparation. The current report reveals that neostigmine, similar to many other drugs, may cause a drug reaction. Co-occurrence of bigeminate premature ventricular contractions, therefore, should be viewed as a herald of possible hemodynamic or cardiac catastrophes. Physicians should hence consider any drugs as a probable cause of drug reaction and should be prepared for necessary resuscitative actions in case it occurs.

## Abbreviations

ANA: antinuclear antibody; C3: complement component 3; C4: complement component 4; PVCs: premature ventricular contractions;

## Competing interests

The authors declare that they have no competing interests.

## Consent

Written informed consent was obtained from the patient and her husband (in the respect of local customs) for publication of this manuscript and accompanying images. A copy of the written consent is available for review by the Editor-in-Chief of this journal. At same time, it should be noted that unfortunately the patient did not agree to undergo C3, C4 and ANA tests.

## Authors' contributions

FY was the responsible anesthesiologist for the patient and scientific coordinator in preparing the case report. MA was the responsible anesthesia resident at the time of patient admission, he performed the following laboratory and skin tests and evaluated their impact on the diagnosis. PK performed searches and prepared the first version of the written case report. All authors read and approved the final version of the manuscript.

## References

[B1] Hunter JM Is it always necessary to antagonize residual neuromuscular block? Do children differ from adults? Br J Anaesth 1996 77 707 709 9014619 10.1093/bja/77.6.707

[B2] Fawcett WJ Neuromuscular block in children Br J Anaesth 1997 78 627 9175986 10.1093/bja/78.5.627

[B3] Fuchs-Buder T Mencke T Use of reversal agents in day care procedures (With special reference to postoperative nausea and vomiting) Eur J Anaesthesiol 2001 18 Suppl 23 23 29 11766248

[B4] Naguib M Lien CA Miller RD Pharmacology of muscle relaxants and their antagonists Textbook of Anesthesiology 2005 1 6 Philadelphia: Elsevier 511 514

[B5] Seed MJ Ewan PW Anaphylaxis caused by neostigmine Anaesthesia 2000 55 574 575 10.1046/j.1365-2044.2000.01125.x 10866721

